# What determines location specificity or generalization of transsaccadic learning?

**DOI:** 10.1167/jov.23.1.8

**Published:** 2023-01-17

**Authors:** Corinna Osterbrink, Arvid Herwig

**Affiliations:** 1 Department of Psychology and Cluster of Excellence Cognitive Interaction Technology, Bielefeld University, Bielefeld, Germany; 2 Department of Psychology, Bielefeld University, Bielefeld, Germany

**Keywords:** transsaccadic learning, eye movements, generalization, location specificity, object specificity

## Abstract

Humans incorporate knowledge of transsaccadic associations into peripheral object perception. Several studies have shown that learning of new manipulated transsaccadic associations leads to a presaccadic perceptual bias. However, there was still disagreement whether this learning effect was location specific (Herwig, Weiß, & Schneider, 2018) or generalizes to new locations (Valsecchi & Gegenfurtner, 2016). The current study investigated under what conditions location generalization of transsaccadic learning occurs. In all experiments, there were acquisition phases in which the spatial frequency (Experiment 1) or the size (Experiment 2 and 3) of objects was changed transsaccadically. In the test phases, participants judged the respective feature of peripheral objects. These could appear either at the location where learning had taken place or at new locations. All experiments replicated the perceptual bias effect at the old learning locations. In two experiments, transsaccadic learning remained location specific even when learning occurred at multiple locations (Experiment 1) or with the feature of size (Experiment 2) for which a transfer had previously been shown. Only in Experiment 3 was a transfer of the learning effect to new locations observable. Here, learning only took place for one object and not for several objects that had to be discriminated. Therefore, one can conclude that, when specific associations are learned for multiple objects, transsaccadic learning stays location specific and when a transsaccadic association is learned for only one object it allows a generalization to other locations.

## Introduction

We have the impression that our visual field is homogeneous, although visual resolution differs drastically between the periphery and the fovea (Strasburger, Rentschler, Jüttner, Juttner, & Jüttner, 2011). One explanation of how this is achieved is that prior knowledge is incorporated into our perception of the entire visual field. For example, the visual system can learn transsaccadic associations (i.e., how an object looks before and after performing a saccadic eye movement). By combining these associations with the actual external peripheral input, perception in the periphery could be enhanced and improve both object recognition and visual search (Herwig & Schneider, 2014; Weiß, Schneider, & Herwig, 2014). Experimentally, it has been shown that, after being exposed to new transsaccadic object changes, peripheral perception is biased in the direction of the learned transsaccadic associations. This effect has been shown in various studies and with multiple object features (Herwig & Schneider, 2014; Köller, Poth, & Herwig, 2020; Osterbrink & Herwig, 2021; Paeye, Collins, Cavanagh, & Herwig, 2018; Valsecchi & Gegenfurtner, 2016). One aspect that is not clear yet though, and that has also been pointed out as an open question in the review article by Stewart, Valsecchi, and Schütz (2020) is the location specificity of this learning process, because two studies that will be described in more detail hereafter have shown seemingly contradictory findings.

Herwig, Weiß, and Schneider (2018) conducted experiments in which participants were exposed to transsaccadic object changes in an acquisition phase and had to report how they perceived peripherally presented objects in a following test phase. More specifically, the spatial frequency of a sinusoidal grating within a “swapped object” defined by shape consistently increased or decreased during the saccade. The spatial frequency of a “normal” object was unchanged. In the following test phase, participants judged the spatial frequency of a peripheral object that was presented at either the previous learning location or a new location. Participants who experienced a “low-to-high” spatial frequency change judged the frequency of the swapped object higher than that of the normal one. In contrast, participants who experienced a “high-to-low” change judged the frequency of the swapped object lower than that of the normal object. Peripheral perception was thus biased by the learned transsaccadic associations. This bias effect was only found at the learned object locations in a retinotopic reference frame, but not when they were presented at the new locations. Furthermore, the study revealed that the effect did not depend on whether the judged object was also at the saccade target location.

In a training session, Valsecchi and Gegenfurtner (2016) let participants compare the size of a foveally and a peripherally presented object. Subsequently, while the subjects performed an eye movement toward the peripheral object, its size was manipulated. This induced a shift in the perceived size in the direction of the size change manipulation. In a post-session, the peripheral object was presented at the same position, as well as at the opposite side of where it appeared in the training session. Again, participants judged the size in comparison to a foveally presented object but did not perform a saccade toward the target afterwards. Results indicated that the transsaccadic learning was not locally specific to the learned location but transferred equally well to the opposite hemifield. The same generalization of transsaccadic learning across hemifields was replicated in a similar experiment in the study by Valsecchi, Cassanello, Herwig, Rolfs, and Gegenfurtner (2020).

The question arises of why transsaccadic learning and the resulting presaccadic perceptual bias effect generalized to a new location in the study by Valsecchi and Gegenfurtner (2016) and Valsecchi et al. (2020), but was location specific in the study by Herwig et al. (2018). From a theoretical viewpoint, both generalization as well as specificity have functional and mechanistic advantages and disadvantages. Generalization allows a quick transfer of transsaccadic learning to new retinotopic locations from only a few examples. In an everyday situation in which one moves dynamically through a scene, objects can appear anywhere on the retina, and it is therefore beneficial to recognize them anywhere and irrespective of where they have previously been detected. This kind of generalization requires less memory capacity. At the same time, more complex computations in the visual system are necessary, which slows down the processing (Dill & Fahle, 1997). Location specificity, on the other hand, requires instance-based learning for specific locations which is highly memory intensive but also very precise. Because it does not require complex computations, processing can be very fast (Dill & Fahle, 1997). The present study investigated under what conditions learned transsaccadic associations generalize to other locations and under what conditions they stay specific to the learned location. By answering this open question, further insights into the mechanism behind the presaccadic perceptual bias effect can be gained. Although all three previous studies introduced transsaccadic feature changes to objects and measured how this affected their peripheral perception, the experimental design differed in many aspects. A few of these differences in the existing literature were addressed in Experiments 2 and 3. Another possible contributing factor of generalization was addressed in Experiment 1 of this study.

First, the learning might have been location specific in the study by Herwig et al. (2018), because only two different saccade target positions were used in the acquisition phase. By expanding the learning to more positions, the visual system could learn to generalize the learned transsaccadic associations to other locations. A similar effect was shown for saccadic adaptation, which is the recalibration of saccade amplitude in response to systematic position shifts of saccade targets. When adaptation took place in multiple directions, it was spatially generalized instead of being spatially selective, as is the case when participants adapt to only one direction (Rolfs, Knapen, & Cavanagh, 2010). Thus, in the first experiment, instead of having only two learning positions, participants experienced transsaccadic changes when saccading to six different target positions. In addition to these six learned positions, peripheral object perception was judged at six new locations. In the study by Herwig et al. (2018), the learning locations were on the horizontal meridian and thus required cardinal saccades. The four new locations in the test phase were shifted above or below the old ones and therefore required oblique saccades. This difference in saccade performance could also be a reason for the hinderance of generalization. In Experiment 1, cardinal and oblique saccades were performed in the acquisition phase and also in the test phase to possibly rule out this explanation, as well.

Second, the difference in feature choice between the studies was addressed. Although transsaccadic learning of the feature spatial frequency seems to be location specific, size as a feature might allow for generalization to new locations. Consequently, Experiment 2 tested whether there is generalization if the feature that observers learn is size instead of spatial frequency in the paradigm of Herwig et al. (2018).

Third, the number of objects for which distinct associations have to be acquired might play a crucial role in the learning effect. The study design by Valsecchi and Gegenfurtner (2016) and Valsecchi et al. (2020) only contained one object (gray patches with smooth edges) whose size perception was tracked over time. In the study design by Herwig et al. (2018) two objects that differed in shape were presented and participants had to distinguish them in order to fulfill the task (looking at both equally often). In the acquisition phase, only the spatial frequency of one of the objects was manipulated and the other, which did not change transsaccadically, served as a baseline condition. Therefore, specific transsaccadic associations were learned for each object and the difference in perception between these two objects served as a measure of the learning effect. Connecting a certain object to a specific transsaccadic change possibly induces a spatially selective form of learning. In comparison, by only experiencing a certain transsaccadic change of an object whose other features are not relevant and which does not have to be discriminated from others might lead to a general form of learning. The presaccadic bias effect due to transsaccadic learning has also been compared with visual perceptual learning, which is the improvement in task performance with practice (Dosher & Lu, 2017; Sagi, 2011). It is often specific to only task-relevant stimuli, features, or locations (Ahissar & Hochstein, 1993; Dosher & Lu, 2017). But, these specificities of perceptual learning can also be eliminated and depend on the exact conditions of the training procedure. Perceptual learning in object recognition has been shown to be specific to the learned object and to also occur in a location-specific manner (Furmanski & Engel, 2000; Gölcü & Gilbert, 2009; Sigman & Gilbert, 2000). Experiment 3 tested whether the number of objects for which distinct associations have to be acquired plays a role in the spatial generalization of transsaccadic learning. Again, the design was similar to the study by Herwig et al. (2018), with the difference that only one object was presented and manipulated in the acquisition phase. Instead of having a “normal” object as a baseline, the perception of the object was tested before and after the acquisition phase. We hypothesized that the transsaccadic learning would generalize to new locations, because it did not occur in a discriminative and object-specific manner.

## Experiment 1: Learning at multiple locations

### Material and methods

#### Participants

Sixteen subjects participated in this experiment (12 females, four males). They were between 20 and 35 years old (*M* = 23.56, *SD* = 4.34). As they were psychology students at Bielefeld University, they received course credit for their participation. All participants had normal or corrected-to-normal vision. Prior to participating, they gave written informed consent. The experiments were approved by the local ethics committee of Bielefeld University and were in accordance with the tenets of the Declaration of Helsinki, except that they were not preregistered.

#### Apparatus

The experiments were conducted in a dimly lit room. The stimuli were presented on a 19-inch CRT monitor with a resolution of 1024 × 768 pixels, corresponding to a physical width of 36 cm and a height of 27 cm. The refresh rate of the monitor was 100 Hz. The eye movements were recorded using a video-based eye tracker (EyeLink 1000; SR Research, Ottawa, ON, Canada) with a sampling rate of 1000 Hz. The right eye was used for monitoring gaze. Participants sat 71 cm away from the monitor, and their head was stabilized by a chin rest. Subjects’ behavioral responses were recorded via a standard keyboard and mouse. In Experiment 1, Experiment Builder software (SR Research) was used for controlling stimulus presentation and response collection.

#### Stimuli

**Figure 1.**
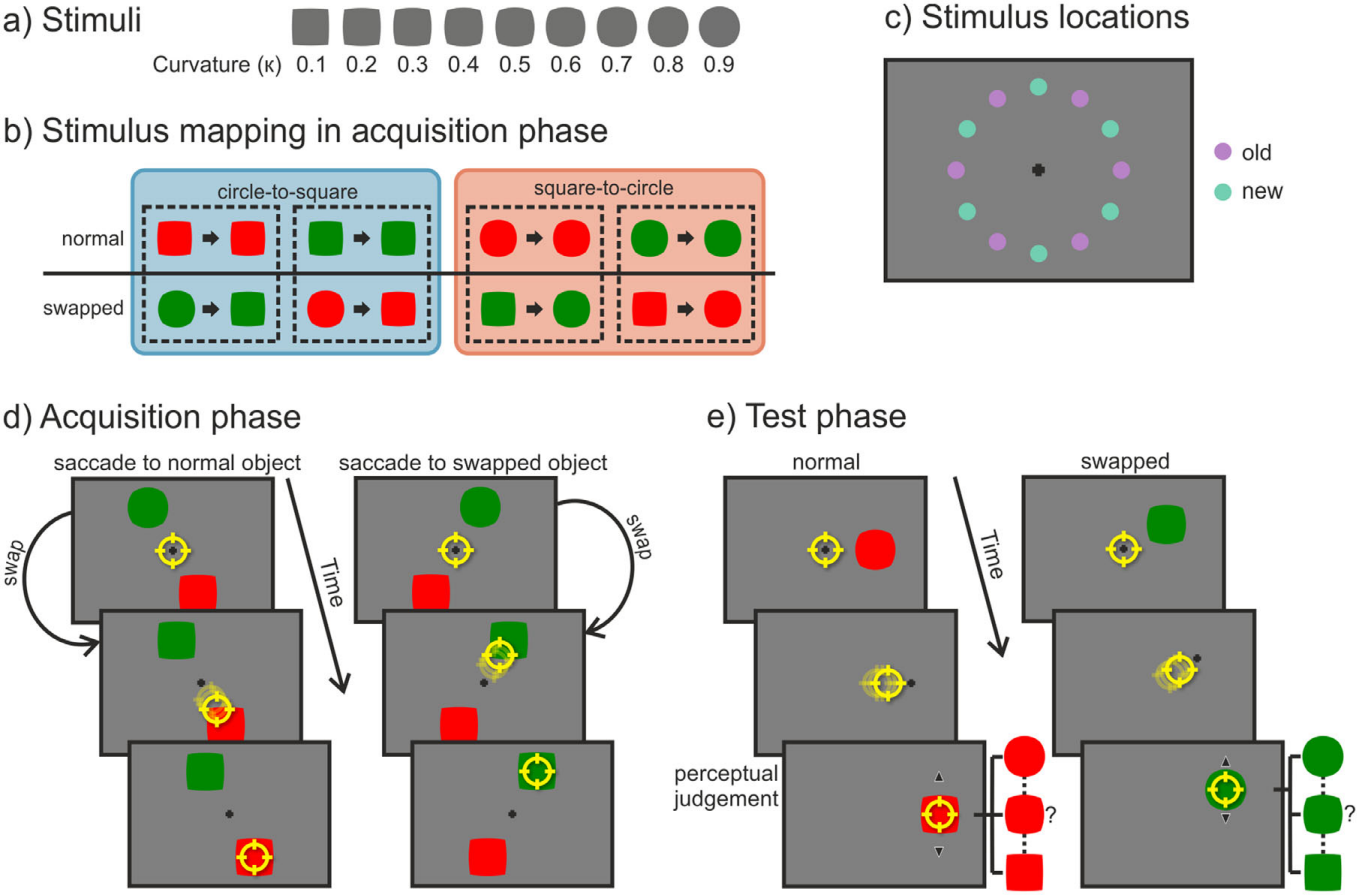
Experiment 1. (a) Shapes and their curvature that were used as stimuli. (b) For each subject (outlined by the dashed lines) there was a fixed mapping between the peripheral (i.e., presaccadic; left of the arrow) and foveal (i.e., postsaccadic; right of the arrow) view of the objects. There was one object with the “normal” status that stayed the same and one object with the “swapped” status that changed either from circle to square (blue box) or square to circle (red box) during the saccade. (c) Possible locations at which the objects could be presented. In the acquisition phase, stimuli were only shown at the six “old” (purple) locations; in the test phase, stimuli were presented at both “old” and “new” (turquoise) locations. (d) Participants could saccade freely to either of two objects that were presented on opposite sides of the fixation cross. During the saccade, the shape (curvature) of the swapped object changed (in this case, to a more square-like shape). (e) Participants had to saccade to the peripheral target, which was replaced by the fixation cross as soon as the eye movement started. After the saccade, the object reappeared with a randomly chosen curvature. Participants could then change the shape of the object via key presses and had to judge how they perceived it presaccadically. Note for (c) and (d) stimuli are not drawn to scale. The yellow circle represents the gaze position.

Stimuli with a luminance of 31 cd/m^2^ were presented on a uniform gray background with a mean luminance of 30 cd/m^2^. A black fixation cross (width and height, 0.3°; line width, 2 pixels) was displayed in the center of the screen. Red and green stimuli of different shapes served as saccade targets. The two-dimensional target shapes (see Figure 1a) were nine intermediate steps of transforming a circle (radius = 0.825°) into a square (side length = 1.46°). The shapes were defined by the curvature of their sides (κ = 0.825°/radius), which range from 0 for the square to 1 for the circle. In the acquisition phase, shapes with curvatures of 0.2 and 0.8 were presented; in the test phase, shapes with curvatures of 0.1, 0.3, 0.5, 0.7, and 0.9 were presented as saccade targets. For giving a response, the curvature of the test shapes could be adjusted between 0.1 and 0.9 in steps of 0.1.

#### Procedure and design

The experiment was divided into an acquisition and a test phase. Each phase started with a nine-point grid calibration of the eye tracker. Each trial in the acquisition phase had the following procedure (see Figure 1d): Participants first had to look at the centrally presented fixation cross for a variable time interval of 500 to 1000 ms. Afterward, two shapes (one green and one red) appeared at exactly opposite sides of the fixation point. The six possible positions of the targets were evenly distributed on an imaginary circle with a radius of 6° around the fixation point (see Figure 1c). The targets appeared with equal frequencies on these fixed six positions, and the sides on which the red and the green target were presented were counterbalanced. The task of each participant was to saccade to either the red or the green object, the choice being their own. They were instructed, though, to look at both objects (object colors) equally often while avoiding fixed viewing patterns such as looking “red–green–red–green.” During the saccade, the curvature of one of the target objects, referred to as the “swapped” object, either increased (from 0.2 to 0.8) or decreased (from 0.8 to 0.2). The curvature of the “normal” object stayed the same and was the same curvature as the postsaccadic swapped object. The presaccadic/peripheral curvature of the two shapes thus differed, but their postsaccadic/foveal curvature was always the same. The mapping of which of the two objects (red or green) changed in curvature and whether the curvature increased or decreased was fixed for each participant, but was counterbalanced among all participants (see Figure 1b). After the saccade ended, the objects stayed visible for an additional 250 ms followed by an intertrial interval of 1500 ms in which only a blank screen was shown. The acquisition phase consisted of 720 trials divided into 10 blocks. After each block of 72 trials, participants received feedback on the screen on the number of times they had looked at each object.

The procedure of the test phase was as follows (see Figure 1e): Participants first had to look at the fixation cross for 500 to 1000 ms. Afterward, a green or red target object with a curvature of 0.1, 0.3, 0.5, 0.7, or 0.9 appeared. In two thirds of the trials, the median curvature of 0.5 was used; in one third of the trials, the other curvatures were presented with equal frequencies as catch trials to ensure that participants would not notice the uniformity of the curvature. The target object was positioned at one of 12 possible locations. Six locations were the old locations from the acquisition phase, and the other six new locations were exactly in between the old ones on the imagined circle (see Figure 1c). The participants’ first task was to saccade to the target object as quickly and accurately as possible. If the saccade had not been started after 350 ms the trial was aborted, and participants received an error message asking them to execute the eye movement more quickly. During the saccade, the object was replaced by a fixation cross. Participants thus never had a foveal view of the object, but only a peripheral one. After 500 ms, an object with the same object color was presented, but with a randomly chosen curvature. The second task was to indicate which object curvature participants had perceived in the periphery. They could go through the different curvatures (0.1 to 0.9 in steps of 0.1) via the up- or downward arrow keys on the keyboard, and they selected their response by pressing Enter on the keyboard. The test phase consisted of 288 trials which were run in six blocks of 48 trials.

#### Data analysis

Saccade onsets and offsets were identified by the EyeLink parser using a velocity criterion of 30°/s and an acceleration criterion of 8000°/s^2^. Single trials were excluded offline from the analysis, if (a) the saccade was anticipatory (saccade latency < 80 ms) (Fischer, Weber, Biscaldi, Aiple, Otto, & Stuhr, 1993; Wenban-Smith & Findlay, 1991); (b) saccade latency was too large (latency > 1000 ms in the acquisition phase or latency > 350 ms in the test phase); (c) the saccade to the target started outside of a 2° radius of the fixation point; (d) the saccade end points were outside of a 3° radius from the center of the target; or (e) the swap of the changed object in the acquisition phase or the removal of the target object in the test phase did not occur during the saccade. Based on the above-mentioned criteria, 12.64% of trials were discarded in the acquisition phase and 8.68% in the test phase.

Statistics were computed in R (R Core Team, 2019) using the packages tidyr and dplyr (Wickham, François, Henry, & Müller, 2019; Wickham & Henry, 2019) for data aggregation and preparation and the package ez (Lawrence, 2022) for the conducted analyses, as well as the package ggplot (Wickham, 2016) for the visualization. The Bayesian analysis was conducted with the R packages BayesFactor (Morey & Rouder, 2018) and bayestestR (Makowski, Ben-Shachar, & Lüdecke, 2019) using default priors. The significance criterion for the analyses of all experiments was set to *p* < 0.05. Statistical *t*-tests were two sided.

### Results

#### Acquisition phase

Participants looked at the normal object in 49.6% of trials and at the swapped object in 50.4% of trials, which is about equally often, *t*(15) = −1.11, *p* = 0.283, *d* = −0.28. The mean saccade latency, averaged over the median per participant, was 257.0 ms (*SD* = 33.7 ms). Averaged over the means of each participant, the mean saccade duration was 44.8 ms (*SD* = 3.6 ms), and the swapping of the object occurred on average 21.9 ms (*SD* = 1.2 ms) after saccade onset.

#### Test phase

Only trials in which an object with the median curvature of 0.5 was shown as saccade target in the periphery were included in the analysis. The catch trials were discarded. First, data were collapsed for each participant among position status, thus across all old positions and across all new positions. A mixed analysis of variance (ANOVA) on the shape judgments (curvature in κ) was run with the within-subjects factors object status in the acquisition phase (normal vs. swapped) and position status (old vs. new), as well as the between-subjects factor change direction (circle to square vs. square to circle). Additionally, a Bayesian ANOVA was conducted. The comparisons of the null model containing only the random factor of participant with all other alternative models containing or not main and interaction effects are expressed as Bayes factors of model comparisons (BF_10_) (see Supplementary Table S1). Moreover, inclusion Bayes factors (BF_incl_), which indicate evidence for including a predictor, were computed by averaging over the “matched” models only (see Supplementary Table S2).

**Figure 2.**
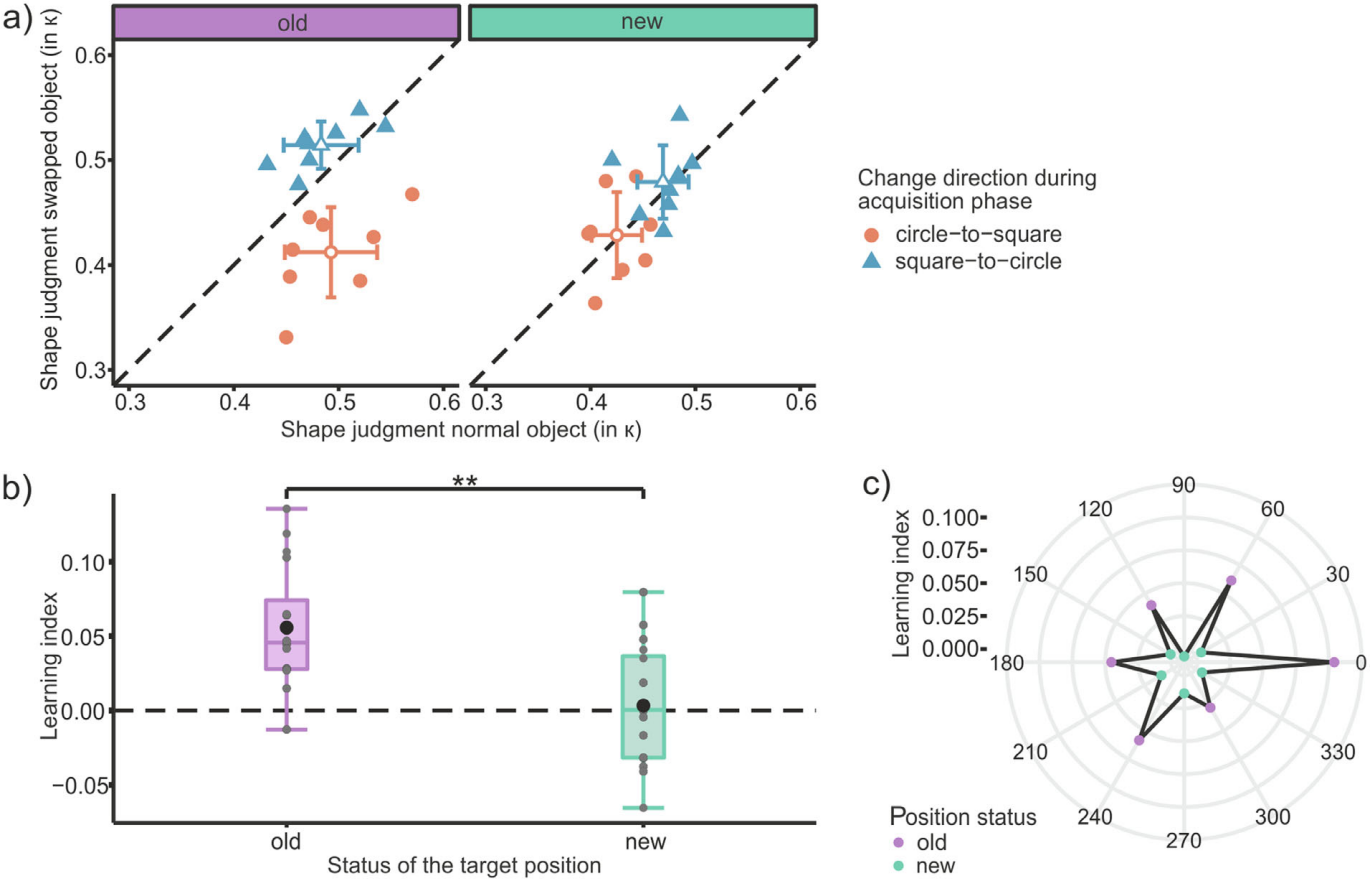
Results of Experiment 1. (a) Mean shape judgments (in curvature κ) of the swapped object as a function of the shape judgment of the normal object for the old (left) and the new (right) target positions. Filled shapes represent data from single participants, and open shapes represent the average across participants. For the latter, the error bars represent the standard deviations of the means. Color and shape distinguish the change direction of the swapped object during the acquisition phase. (b) Boxplots of learning indices (i.e., judgment differences between normal and swapped object) for the old and new target positions. Positive values indicate a judgment bias toward the associated foveal image. Gray dots indicate the learning index for each participant; the black circle shows the mean learning index. (c) Learning indices averaged over all participants for each target position. The color distinguishes between old and new target positions.

The ANOVAs revealed a significant main effect of change direction, *F*(1, 14) = 12.20, *p* = 0.004, η_G_^2^ = 0.34, BF_incl_ = 9.94, and position status *F*(1, 14) = 18.82, *p* < 0.001, η*_G_*^2^ = 0.13, BF_incl_ = 18.58. Furthermore it revealed an interaction effect of change direction × object status, *F*(1, 14) = 18.51, *p* < 0.001, η*_G_*^2^ = 0.17, BF_incl_ = 101.92, as well as position status × object status *F*(1, 14) = 6.10, *p* = 0.027, η*_G_*^2^ = 0.06, BF_incl_ = 1.91. Most importantly the three-way interaction of change direction × position status × object status was also significant, *F*(1, 14) = 16.87, *p* = 0.001, η*_G_*^2^ = 0.14, BF_incl_ = 106.75. The model containing this three-way interaction was also the one with the highest Bayes factor (BF_10_ = 169,041.60). It also includes all other main and interaction effects due to the principle of marginality (Nelder, 1977). The effect of the three-way interaction can be nicely seen in Figure 2a, in which average shape judgments of the swapped object are plotted against the normal object for each participant. When targets were presented at the old positions (see left plot), one can see a clear separation of points by the equality line based on the change direction in the acquisition phase. Participants whose swapped object changed from a circle to a square judged the curvature of the swapped object lower (more square-like) than that of the normal one. Participants whose object changed from a square to a circle judged the curvature of the swapped object higher (rounder) than that of the normal one. This separation cannot be seen at the new positions (see Figure 2a, right plot). The normal and swapped object are judged similarly here. The main effect of object status was not significant, *F*(1, 14) = 1.72, *p* = 0.211, η*_G_*^2^ = 0.02, BF_incl_ = 0.383, as well as the interaction effect of change direction × position status, *F*(1, 14) = 0.01, *p* = 0.931, η*_G_*^2^ < 0.01, BF_incl_ = 0.334.

A learning index was computed as a measure for the presaccadic perceptual bias, thus for the effect of the transsaccadic learning on the presaccadic shape judgments (Herwig & Schneider, 2014; Osterbrink & Herwig, 2021; Paeye et al., 2018). For this, the average shape judgment of the swapped object was subtracted from the shape judgment of the normal object in the circle-to-square group, and the normal shape judgment was subtracted from the swapped shape judgment in the square-to-circle group. A positive learning index thus indicated a perceptual bias in the direction of the associated foveal image.

The learning indices grouped by the position status and calculated for each participant can be seen in Figure 2b. The learning index at the old positions is significantly higher than at the new positions, *t*(15) = 3.55, *p* = 0.003, *d* = 0.89, BF_10_ = 15.313. Testing whether the learning index was significantly greater than zero showed that this was only the case at the old positions, *t*(15) = 5.44, *p* < 0.001, *d* = 1.36, BF_10_ = 398.101, but not at the new positions, *t*(15) = 0.33, *p* = 0.748, *d* = 0.08, BF_10_ = 0.268. The learning index was furthermore calculated for each position separately, and the average across all participants is depicted in Figure 2c. It can clearly be seen in the star-shaped results that, exactly at the old positions, the learning index is high, and at the new positions the learning index is lower. In the test phase, the mean of the median saccade latency per participant was 177.6 ms (*SD* = 23.5 ms). The mean saccade duration was 44.7 ms (*SD* = 4.0 ms) and the removal of the target object occurred on average 22.0 ms (*SD* = 1.1 ms) after saccade onset.

### Discussion

Experiment 1 investigated whether generalization of transsaccadic learning to new locations could occur when learning takes place at multiple locations. The results reveal a significant three-way interaction in the ANOVA (change direction × position status × object status), as well as a significant learning index at the old positions but not at the new positions. This indicates clearly that transsaccadic learning did not transfer to new locations but instead was only found at the old locations. Only at the locations where transsaccadic learning occurred in the acquisition phase was perception biased in the direction of the transsaccadic change: Participants from the circle-to-square group perceived the swapped object as being more square-like compared with the normal object, and participants from the square-to-circle group perceived the swapped object to be rounder than the normal object.

## Experiment 2: Testing with size as the feature

### Material and methods

#### Participants

Twenty subjects participated in this experiment (eight females, 12 males). They were between 18 and 52 years old (*M* = 24.80, *SD* = 7.84). The participation criteria and ethical standards were the same as in Experiment 1.

#### Apparatus

The same set-up as in Experiment 1 was used. The only difference was that PsychoPy3 (Peirce, 2007) was used for stimulus presentation and response collection.

#### Stimuli

Stimuli were presented on a uniform gray background with a mean luminance of 61 cd/m^2^. A black fixation cross (width and height, 0.3°; line width, 2 pixels) was displayed in the center of the screen. A black circle and square served as saccade targets in the acquisition and test phases. As saccade targets, they had a size of either 1.35° or 1.65°, which refers to the diameter of the circle and the edge length of the square. In the test phase, the circle and square additionally were presented as test objects for giving a response and could be adjusted among the following sizes: 1.2°, 1.35°, 1.5°, 1.65°, and 1.8°.

#### Procedure and design

**Figure 3.**
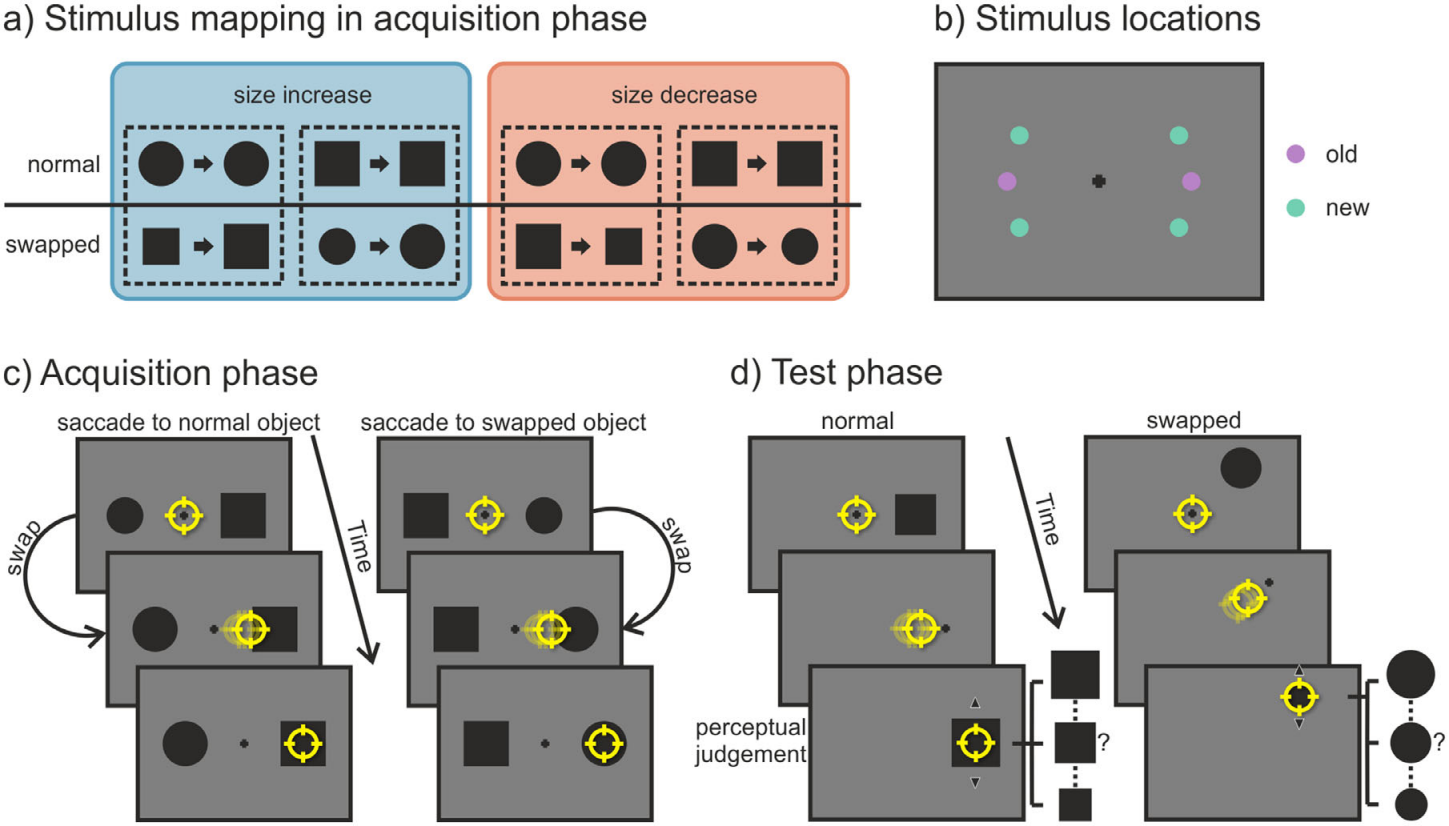
Experiment 2. (a) For each subject (outlined by the dashed lines) there was a fixed mapping between the peripheral (i.e., presaccadic; left of the arrow) and foveal (i.e., postsaccadic; right of the arrow) view of the objects. There was one object with the “normal” status that stayed the same and one object with the “swapped” status that either increased in size (blue box) or decreased in size (red box) during the saccade. (b) Possible locations at which the objects could be presented. In the acquisition phase, stimuli were only shown at the two “old” (purple) locations; in the test phase, stimuli were presented at both “old” and “new” (turquoise) locations. (c) Participants could saccade freely to either of two presented objects. During the saccade, the size of the swapped object changed (in this case increased). (d) Participants had to saccade to the peripheral target, which was replaced by the fixation cross as soon as the eye movement started. After the saccade, the object reappeared with a randomly chosen size. Participants could then change the size of the object via the mouse wheel and had to judge how they perceived it presaccadically. Note that for (c) and (d) stimuli are not drawn to scale. The yellow circle represents the gaze position.

Again, the experiment was divided into an acquisition and a test phase, each starting with a nine-point grid calibration of the eye tracker. In the acquisition phase (see Figure 3c), participants first had to look at the centrally presented fixation cross for a variable time interval of 500 to 1000 ms. Subsequently, a black circle appeared on the horizontal meridian 6° left of the center, and a black square appeared 6° right of the center or vice versa. The sides on which they were presented were counterbalanced. The participants’ task was to saccade to either one of the shapes, the choice being their own. They were instructed, though, to look at both shapes equally often while avoiding fixed viewing patterns such as looking “square–circle–square–circle” or “left–right–left–right.” During the saccade, one of the target objects, referred to as the “swapped” object, increased (from 1.35° to 1.65°) or decreased (from 1.65° to 1.35°) in size. The size of the “normal” object stayed the same and was the size of the postsaccadic swapped object. The presaccadic/peripheral size of the two shapes thus differed, but their postsaccadic/foveal size was always the same. The mapping of which of the two shapes (circle/square) changed in size and whether the size increased or decreases was fixed for each participant but was counterbalanced among all participants (see Figure 3a). After the saccade ended, the objects stayed visible for an additional 250 ms followed by an intertrial interval of 1500 ms during which only a blank screen was shown. The acquisition phase consisted of 240 trials divided into five blocks. After each block of 48 trials, participants received feedback on the screen on the number of times they had looked at each object shape.

In the test phase (see Figure 3d), participants first had to look at the fixation cross for 500 to 1000 ms. Afterward, a target object (a circle or square with a size of 1.35° or 1.65°) appeared at one of six possible locations. Two locations on the horizontal meridian were the “old” locations from the acquisition phase. The other four “new” locations were also 6° away from the central fixation cross but were at an angular separation of 30° above or below the horizontal meridian (see Figure 3b). The participants’ first task was to saccade to the target object as quickly and accurately as possible. If the saccade had not been started after 350 ms, the trial was aborted, and participants received an error message asking them to execute the eye movement more quickly. During the saccade, the object was replaced by a fixation cross. Thus, participants only had a peripheral view of the object, but never a foveal one. After 500 ms, the same object shape was presented again at the position of the target object, but with a randomly chosen size (1.2°, 1.35°, 1.5°, 1.65°, or 1.8°). The second task was to indicate which object size participants had perceived in the periphery. They could go through the different sizes (1.2°, 1.35°, 1.5°, 1.65°, or 1.8°) via the mouse wheel, and they selected their response by keypress on the keyboard. The test phase consisted of 256 trials, which were run in eight blocks of 32 trials. After that, all aborted trials were repeated in a randomized order.

#### Data analysis

The same criteria for saccade identification and trial exclusions as in Experiment 1 were applied. Based on these latter criteria, 5.06% of trials were discarded in the acquisition phase and 9.17% in the test phase. The statistical software and packages used for the analysis were also identical to Experiment 1.

### Results

#### Acquisition phase

Participants looked at the normal object in 50.1% of trials and at the swapped object in 49.9% of trials, which is about equally often, *t*(19) = 0.27, *p* = 0.791, *d* = 0.06. Averaged over the median per participant, the mean saccade latency was 221.2 ms (*SD* = 50.8 ms). The mean saccade duration was 43.3 ms (*SD* = 4.9 ms), and the swapping of the object occurred on average 21.7 ms (*SD* = 0.7 ms) after saccade onset.

#### Test phase

First, as a dependent variable, the judgment error was calculated as the absolute difference between the judged and the presented size of the target object. This was necessary as two different object sizes were presented and judgments of these were analyzed compared with Experiment 1 in which only the judgments of the median shape were analyzed. The judgment errors were collapsed among position status; thus, data from all old positions and all new positions were averaged for each participant. A mixed ANOVA on the judgment errors was run with the within-subjects factors object status in the acquisition phase (normal vs. swapped) and position status (old vs. new), as well as the between-subjects factor change direction (increase vs. decrease). A Bayesian ANOVA was also conducted (see Supplementary Tables S3 and S4).

**Figure 4.**
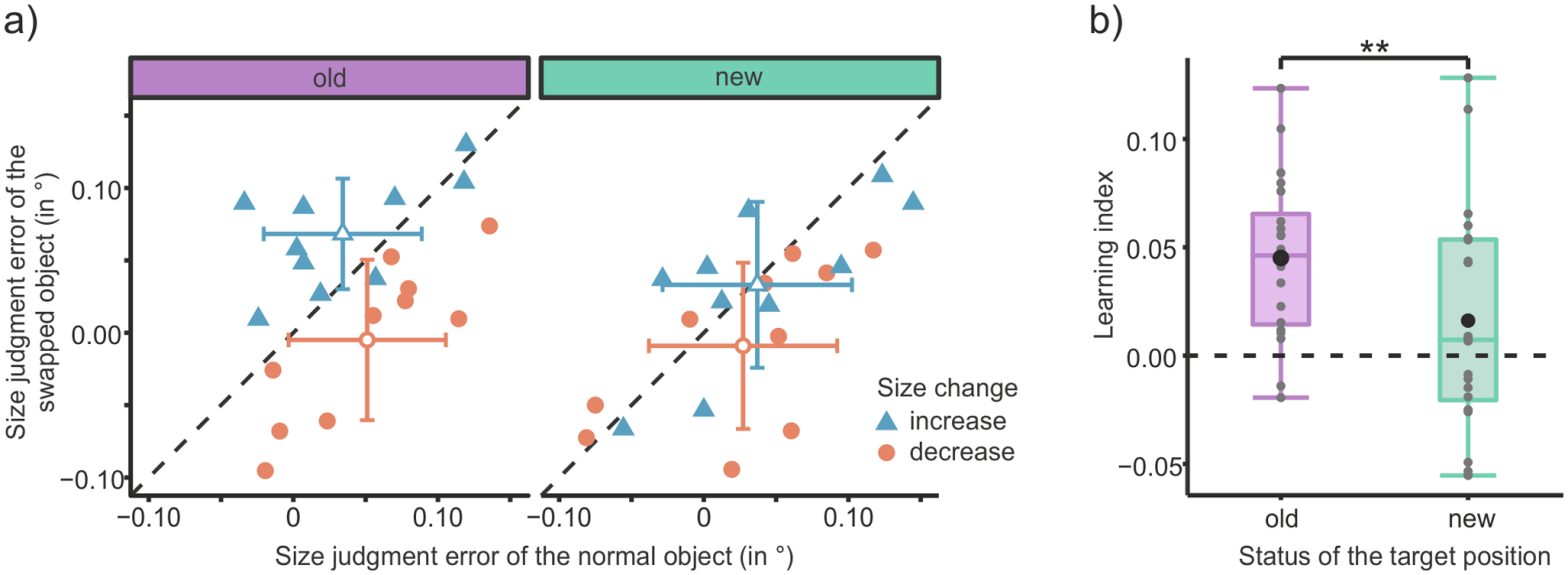
Results of Experiment 2. (a) Mean size judgment errors (°) of the swapped object as a function of the size judgment error of the normal object for the old (left) and the new (right) target positions. Filled shapes represent data from single participants, and open shapes represent the average across participants. For the latter, the error bars represent the standard deviations of the means. Color and shape distinguish the size change direction of the swapped object during the acquisition phase. (b) Boxplots of learning indices (i.e., judgment differences between normal and swapped object) for the old and new target positions. Positive values indicate a judgment bias toward the associated foveal image. Gray dots indicate the learning index for each participant; the black circle shows the mean learning index.

The analysis revealed a significant main effect of position status, *F*(1, 18) = 7.18, *p* = 0.015, η*_G_*^2^ = 0.02, BF_incl_ = 2.01, and an interaction effect of change direction × object status *F*(1, 18) = 12.38, *p* = 0.002, η*_G_*^2^ = 0.08, BF_incl_ = 648.29. Furthermore, the three-way interaction of position status × change direction × object status was also significant, *F*(1, 18) = 10.60, *p* = 0.004, η*_G_*^2^ = 0.02, BF_incl_ = 2.16. All other effects were not significant: change direction, *F*(1, 18) = 1.43, *p* = 0.247, η*_G_*^2^= 0.06, BF_incl_ = 0.754; object status, *F*(1, 18) = 3.17, *p* = 0.092, η*_G_*^2^ = 0.02, BF_incl_ = 1.23; change direction × position status, *F*(1, 18) = 0.04, *p* = 0.849, η*_G_*^2^ < 0.01, BF_incl_ = 0.295; and position status × object status, *F*(1, 18) = 1.04, *p* = 0.322, η*_G_*^2^ < 0.01, BF_incl_ = 0.371. The model of most interest to our hypothesis, the one containing the three-way interaction, was not the model with the highest Bayes factor. The best model (object status + position status + change direction + object status: change direction) was around four times more likely than it. Looking at Figure 4a, in which the size judgment error of the swapped object is plotted against that of the normal object, the three-way interaction can be seen nicely. Only when the target object was presented at the old positions (left plot) is a clear separation of points according to the group factor of change direction visible. The swapped object was perceived bigger than the normal object if the swapped object increased in size during the acquisition phase. In contrast, the swapped object was perceived smaller than the normal object if the swapped object decreased in size during acquisition. This effect is not visible for target objects presented at new positions (right plot).

As in Experiment 1, a learning index was computed. This time, the judgment error of the normal object was subtracted from that of the swapped object in the size-increase group and vice versa for the size-decrease group. The learning index is significantly higher at the old positions than at the new positions (see Figure 4b), *t*(19) = 3.25, *p* = 0.004, *d* = 0.73, BF_10_ = 10.64. At the old positions there is extreme evidence that the learning index is greater than zero, *t*(19) = 5.35, *p* < 0.001, *d* = 1.20, BF_10_ = 685.98. At the new positions the learning index is not significantly different from zero, *t*(19) = 1.38, *p* = 0.184, *d* = 0.31, but the evidence in favor of the null hypothesis is only anecdotal (BF_10_ = 0.527). The mean of the median saccade latency per participant in the test phase was 153.7 ms (*SD* = 23.2 ms). The mean saccade duration was 41.3 ms (*SD* = 3.5 ms), and the removal of the target object occurred on average 21.7 ms (*SD* = 0.7 ms) after saccade onset.

### Discussion

Experiment 2 investigated whether there is generalization to new locations when the feature that observers learn transsaccadically is size. As in Experiment 1, there was a significant three-way interaction in the ANOVA (position status × change direction × object status) and a significant learning index at the old positions, but not at the new position. This indicates that only at the old positions was there a presaccadic perceptual bias in the direction of the transsaccadic learning in the acquisition phase: Participants perceived the swapped object as bigger than the normal object if they were subjected to a size increase in the acquisition phase. In contrast, they perceived the swapped object as smaller than the normal object if they were subjected to a size decrease in the acquisition phase. This perceptual bias was not evident at the new positions.

## Experiment 3: Testing for the number of objects

### Material and methods

#### Participants

Sixteen subjects participated in this experiment (10 females, six males). They were between 18 and 35 years old (*M* = 24.81, *SD* = 4.07). Subjects received monetary reimbursement or course credit for their participation. The participation criteria and ethical standards were the same as in Experiment 1.

#### Apparatus

The same set-up as in Experiment 1 was used. As in Experiment 2, PsychoPy3 (Peirce, 2007) was used for stimulus presentation and response collection.

#### Stimuli

The stimuli were identical to the ones in Experiment 2 with the following differences: Only a black square was used as the saccade target and no circle. The test objects for giving a response could be adjusted between 1.05° and 1.95° in steps of 0.1°. The range was thus a bit bigger and the steps a bit smaller.

#### Procedure and design

**Figure 5.**
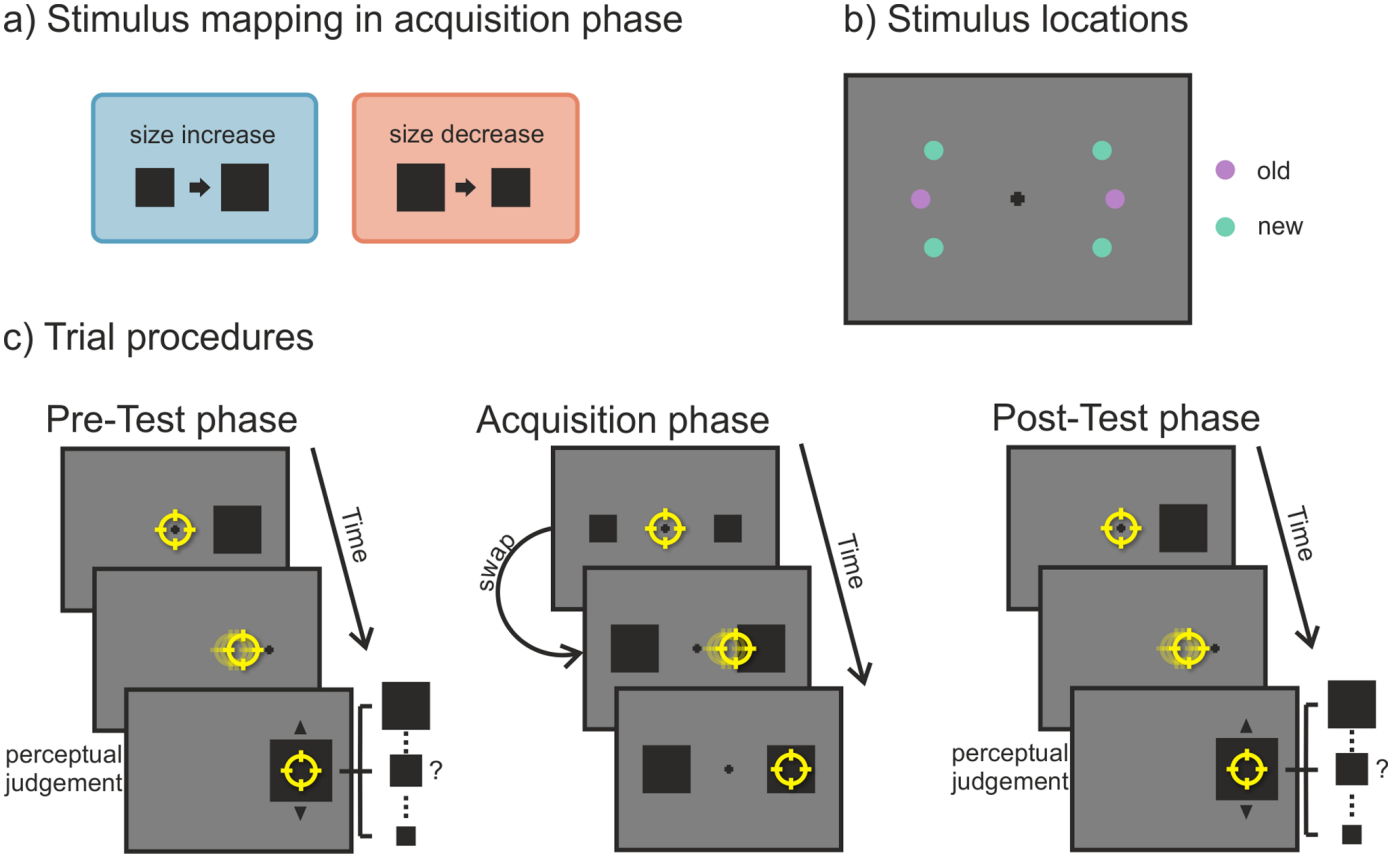
Experiment 3. (a) For each subject the square could either increase or decrease in size during the acquisition phase. (b) Possible locations at which the object could be presented. In the acquisition phase, stimuli were only shown at the two “old” (purple) locations; in the test phases, stimuli were presented at both “old” and “new” (turquoise) locations. (c) Trial procedure in the acquisition phase: Participants could saccade freely to either of two presented objects. During the saccade, the size of the objects changed (in this case increased). Trial procedure in the pre-test and post-test phases: Participants had to saccade to the peripheral target, which was replaced by the fixation cross as soon as the eye movement started. After the saccade, the object reappeared with a randomly chosen size. Participants could then change the size of the object via the mouse wheel and had to judge how they perceived it presaccadically. Note that for (c) stimuli are not drawn to scale. The yellow circle represents the gaze position.

The experiment was divided into a pre-test phase, an acquisition phase, and a post-test phase. Each phase started with a nine-point grid calibration of the eye tracker. The basic trial procedures were the same as in Experiment 2 with the following modifications (see Figure 5c): First, a pre-test phase was performed. A black square with a size of 1.35° or 1.65° was presented as a possible saccade target at one of six possible locations (see Figure 5b). Participants had to look at this target, which disappeared during the saccade. Then, a square with a randomly chosen size (between 1.05° and 1.95° in steps of 0.1°) was presented. Participants could go through these different sizes (between 1.05° and 1.95° in steps of 0.1°) to indicate which size they had perceived in the periphery. The test phase consisted of 128 trials that were run in four blocks of 32 trials. After that, all the aborted trials were repeated in a randomized order.

The acquisition phase was then performed. After centrally fixating, two squares appeared. The participants’ task was to saccade to either one of them but to look at the left and right side equally often while avoiding fixed viewing patterns. During the saccade, both squares either increased or decreased in size. This change direction was fixed for each participant but counterbalanced between participants (see Figure 5a). The acquisition phase consisted of 240 trials divided into five blocks. After each block of 48 trials, participants received feedback on the screen on the number of times they had looked at the right and left side. Afterward, a post-test phase was conducted that was exactly the same as the pre-test phase.

#### Data analysis

The same criteria for saccade identification as well as for trial exclusions as in Experiment 1 were applied. Based on these latter criteria, 5.44% of trials were discarded in the acquisition phase and 7.70% in the test phase. The statistical software and packages used for the analysis were also identical to Experiment 1.

### Results

#### Acquisition phase

Participants followed the task and looked at the left and the right side equally often, *t*(15) = 0.99, *p* = 0.338, *d* = 0.25. The average of the median saccade latency of each participant was 206.8 ms (*SD* = 34.9 ms). The mean saccade duration was 46.3 ms (*SD* = 5.1 ms), and on average the swapping of the object occurred 19.7 ms (*SD* = 3.5 ms) after saccade onset.

#### Pre-test and post-test phases

As in Experiment 2, the size judgment error, and thus the absolute difference between the judged and the presented object size, was taken as the dependent variable. Similarly, the data were again collapsed among positions status (old vs. new). A mixed ANOVA on the judgment errors was run with the within-subjects factors test phase (pre- vs. post-) and position status (old vs. new), as well as the between-subjects factor size change direction (increase vs. decrease).

The ANOVA revealed two significant interaction effects: first, the interaction of change direction × test phase, *F*(1, 14) = 7.64, *p* = 0.015, η*_G_*^2^ = 0.03, and, second, the interaction of position status × test phase, *F*(1, 14) = 5.05, *p* = 0.041, η*_G_*^2^ < 0.01. All other effects were not significant: change direction, *F*(1, 14) = 0.94, *p* = 0.349, η*_G_*^2^ = 0.06; position status, *F*(1, 14) = 4.57, *p* = 0.051, η*_G_*^2^ < 0.01; test phase, *F*(1, 14) = 0.13, *p* = 0.720, η*_G_*^2^ < 0.01; change direction × position status, *F*(1, 14) = 0.22, *p* = 0.647, η*_G_*^2^ < 0.01; or change direction × position status × test phase, *F*(1, 14) = 0.74, *p* = 0.405, η*_G_*^2^ < 0.01.

Furthermore, a Bayesian ANOVA was run (see Supplementary Tables S5 and S6). Looking at the inclusion Bayes factors, one can see that the only predictor that shows evidence for its inclusion is the interaction change direction × test phase (BF_incl_ = 165.33). For all other predictors, there is anecdotal to moderate evidence against including them. Looking at the model comparison, one can see that the model containing exactly this interaction has the highest Bayes factor (BF_10_ = 30.93). Due to the principle of marginality, it also includes the respective main effects (test phase + change direction + test phase: change direction). This model is 15 times more likely than the model including the three-way interaction in question.

**Figure 6.**
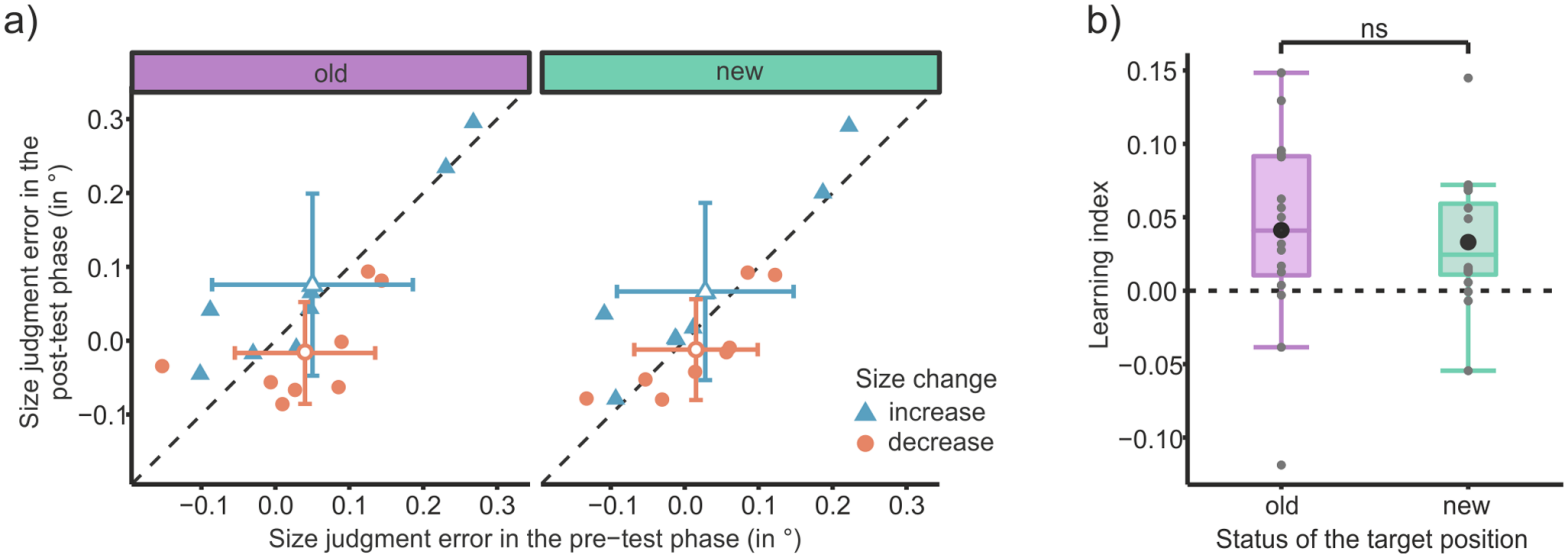
Results of Experiment 3. (a) Mean size judgment errors (°) in the post-test phase as a function of the size judgment error in the pre-test phase for the old (left) and the new (right) target positions. Filled shapes represent data from single participants, and open shapes represent the average across participants. For the latter, the error bars represent the standard deviations of the means. Color and shape distinguish the size change direction during the acquisition phase. (b) Boxplots of learning indices (i.e., judgment differences between pre- and post-test phases) for the old and new target positions. Positive values indicate a judgment bias toward the associated foveal image. Gray dots indicate the learning index for each participant; the black circle shows the mean learning index.

The significant interaction effect for both the old and the new target positions can be seen in Figure 6a, although not as distinctly as in the previous experiments. If the size of the object increased in the acquisition phase, then participants perceived the object bigger in the post-test phase than in the pre-test phase. Conversely, they perceived it as smaller in the post-test phase than in the pre-test phase if the size of the object decreased in size in the acquisition phase.

As in the other experiments, a learning index was calculated. This time, the judgment error in the post-test phase was subtracted from the judgment error in the pre-test phase for the group that experienced a size decrease in the acquisition phase. The opposite subtraction was performed for the size increase group. As is depicted in Figure 6b, the learning index did not differ significantly between the old and new target positions, *t*(15) = 0.76, *p* = 0.458, *d* = 0.19, BF_10_ = 0.329. Moreover, the learning index was significantly higher than zero at old positions, *t*(15) = 2.50, *p* = 0.025, *d* = 0.62, BF_10_ = 2.637, and new positions, *t*(15) = 2.95, *p* = 0.010, *d* = 0.74, BF_10_ = 5.544. Averaged over the medians of all participants, the mean saccade latency was 160.1 ms (*SD* = 28.9 ms). On average, the saccade lasted 42.6 ms (*SD* = 3.9 ms), and removal of the objects occurred after 21.8 ms (*SD* = 0.6 ms).

### Discussion

Experiment 3 investigated whether generalization of transsaccadic learning is conditional on an object-specific learning mode. Instead of learning two object-specific transsaccadic associations, participants only had to learn one. Perception was tested before and after the acquisition phase. As in the previous experiments, there was a significant presaccadic perceptual bias effect, as the significant interaction of change direction × test phase in the ANOVA shows. Thus, participants perceived the object as bigger in the post-test compared with the pre-test phase if they experienced a size increase in the acquisition phase, and they perceived it as smaller if they experienced a size decrease. But, in contrast to the first experiments, the three-way interaction (change direction × position status × test phase) in the ANOVA was not significant. Furthermore, the learning indices at the old and the new positions did not differ significantly, and they were both significantly greater than zero. This indicates that there was a transfer of the transsaccadic learning to new positions.

## General discussion

Prior knowledge in form of transsaccadic associations shapes peripheral perception. When objects are manipulated during the saccade, peripheral perception is biased in the direction of the learned transsaccadic change. This peripheral perceptual bias due to transsaccadic learning has been shown in several studies (Bosco, Lappe, & Fattori, 2015; Bosco, Rifai, Wahl, Fattori, & Lappe, 2020; Herwig & Schneider, 2014; Herwig, Weiß, & Schneider, 2015; Herwig et al., 2018; Köller et al., 2020; Osterbrink & Herwig, 2021; Osterbrink, Recker, & Herwig, 2022; Paeye et al., 2018; Valsecchi & Gegenfurtner, 2016; Valsecchi et al., 2020). Whether these transsaccadic object associations are transferred to new locations is still under debate. Although Valsecchi and Gegenfurtner (2016) showed that the learning can be transferred to the horizontal mirror location, Herwig et al. (2018) showed that they are location specific in a retinotopic reference frame. The current study thus investigated under what circumstances generalization to new locations occurs. Learning transsaccadic changes at multiple target locations did not lead to a transfer of the learning effect to new locations (Experiment 1). Generalization of transsaccadic changes did not automatically occur for size as a feature (Experiment 2). Instead, generalization of transsaccadic learning to new locations seems to depend on object specificity. When only one new transsaccadic association is learned, it can be transferred to new locations (Experiment 3). When multiple associations are learned specific to certain objects, learning seems to be location specific.

### Learning at multiple locations

Experiment 1 investigated whether transsaccadic learning generalizes to new locations when learning occurs at multiple locations instead of only two as in Herwig et al. (2018). Dill and Fahle (1997) also suggested that multiple position-specific learning events might be necessary before perceptual learning, in this case of pattern discrimination, is independent of retinal location. Rolfs et al. (2010) also showed that, by inducing saccadic adaptation in all directions via a quasi-random walk, it was not spatially selective but global instead. To test whether the same could be true for transsaccadic learning, participants made saccades to six different target locations. Meanwhile, the shape of one of the two presented objects changed. In the following test phase, target objects were presented at these six old positions and at six new positions. Results revealed a significant transsaccadic learning effect only at the old positions. That is, exactly at the locations at which transsaccadic learning took place, there was a perceptual bias in the direction of the transsaccadic association. The shape of the swapped object was perceived as more curved (rounder) for the group that experienced a square-to-circle change, and it was perceived as less curved (more square-like) in the circle-to-square group. The learning index, representing the strength of the perceptual bias, at the new positions was not significant. In summary, transsaccadic learning was location specific and did not generalize to new locations even though learning occurred at multiple learning positions. One constraint of our study is, of course, that, contrary to Rolfs et al. (2010), the number of learning locations was relatively limited and might still not be high enough to lead to location generalization. Nevertheless, we replicated the finding that transsaccadic learning is highly location specific. In future studies, one could adapt the design of Rolfs et al. (2010) and perform saccades to random locations on the screen while transsaccadically manipulating object features.

### Feature peculiarities

Experiment 2 tested whether location generalization only occurs for specific features. Bosco et al.(2015) showed that transsaccadic changes in object size lead to adaptations of saccade amplitude, as well as perceived object size. A later study revealed that the induced change in size perception is independent from the saccadic amplitude change (Bosco et al., 2020). In the study by Valsecchi and Gegenfurtner (2016), which also manipulated and tested the feature “object size,” location generalization of this learning effect occurred. In the study by Herwig et al. (2018), spatial frequency was used as a feature, and learning was location specific. Therefore, in the current study, the experimental design of Herwig et al. (2018) was used but, instead of changing spatial frequency, object size was manipulated. Again, the perceptual bias effect due to the transsaccadic feature change could be shown, but only at those locations where learning had occurred. The learning effect did not transfer to new locations. Consequently, one can conclude that it was not size as a feature that led to a spatial generalization in the study by Valsecchi and Gegenfurtner (2016).

### Number of object specific associations

Experiment 3 investigated whether the number of objects for which distinct associations have to be acquired plays a role in the location generalization of transsaccadic learning. Herwig et al. (2018) presented two objects (“normal” and “swapped” object) that had to be differentiated and for which specific transsaccadic associations had to be learned. In the current study, as was the case in Valsecchi and Gegenfurtner (2016) and Valsecchi et al. (2020), a new transsaccadic association (a size change) was learned for only one object. As we hypothesized, transsaccadic associations transferred to new locations in this case. A presaccadic perceptual bias in the direction of the transsaccadic manipulation was found at the old locations, where the learning had occurred, but also at new locations.

When multiple objects are presented in the acquisition phase that must be differentiated to fulfill the task, participants can learn new transsaccadic associations for each of them. This object-specific learning has also been shown in the study by Osterbrink and Herwig (2021), in which transsaccadic associations between individual balls and fruits were established. When only one object is presented whose features and overall identity are practically irrelevant for the task at hand, the transsaccadic learning might be more general and not specific to the object. We suggest that this lack of object specificity in the learning process when only one transsaccadic association is learned for a single object is the reason why a transfer to new locations is possible. On the other hand, when specific associations are learned for multiple objects, the presaccadic perceptual bias stays location specific.

The experiments that showed location specificity (Experiments 1 and 2) (Herwig et al., 2018) and the experiments that showed location generalization (Experiment 3) (Valsecchi & Gegenfurtner, 2016; Valsecchi et al., 2020) differ in the number of the objects used (one vs. two objects) and the uniformity of the transsaccadic change (learning one transsaccadic association vs. two different transsaccadic associations). From these studies, it is not possible to differentiate which of the two aspects (lack of object discrimination because there is only one object or the uniformity in the transsaccadic change) is the relevant factor in determining location generalization. We would argue that, when the visual system notices deviating transsaccadic changes, it automatically connects each transsaccadic association to the particular object. As a result of this object specificity of the association, it also seems to be location specific. It would be interesting to see in a future experiment whether learning a uniform transsaccadic change for different objects also leads to location generalization.

The underlying reason for the location specificity in the case of multiple object learning might be an object-location binding. Shafer-Skelton, Kupitz, and Golomb (2017) investigated transsaccadic object-location binding via the spatial congruency bias. This describes the effect that people are more likely to judge two objects as the same identity if they are presented at the same location compared with different locations. Their results indicated that the spatial congruency bias was preserved transsaccadically in retinotopic coordinates. Thus, object location seems to act as an indirect link (i.e., as a pointer or “object file”) during object recognition (Kahneman, Treisman, & Gibbs, 1992; Shafer-Skelton et al., 2017). This binding of object identity and retinotopic location also fits well to the results of Herwig et al. (2018), which showed that transsaccadic learning was specific to the learned retinotopic location if it is bound to a specific object.

Another possible explanation for the effect in our experiment would be that, because there is only a single object, participants simply learn the size of the object. For our experiment, this would in theory be conceivable, but we would argue that it is not the reason for the generalization. In the experiment by Valsecchi et al. (2020), in which the relative transsaccadic size changes were manipulated, the absolute sizes of the peripheral target object were randomly chosen among five possible ones. Thus, simply learning the size of the object was not possible. Nevertheless, the relative transsaccadic change was learned and transferred to the mirror location.

The presaccadic bias effect due to transsaccadic learning has also been compared with visual perceptual learning. The latter has been classically described to be specific to the learned stimuli, task, or retinal location (Dill & Fahle, 1997; Goldstone, 1998). Early studies therefore suggested that perceptual learning takes place in the primary visual cortex. More recent research has challenged these findings by showing that retinotopic specificity depends on the particularities of the training procedure (Hung & Seitz, 2014). The transfer of perceptual learning to different retinal locations was, for example, enabled by a double training procedure (Xiao, Zhang, Wang, Klein, Levi, & Yu, 2008) or by including a brief pre-test at the transfer locations (Zhang, Xiao, Klein, Levi, & Yu, 2010). Consequently, different models that involve higher brain areas have been suggested to explain perceptual learning and its specificity or generalization: rule-based reweighting of inputs (Zhang, Zhang, Xiao, Klein, Levi, & Yu, 2010), blockage of top–down influences (Zhang, Cong, Song, & Yu, 2013), or the improvement of brain networks that integrate processes (e.g., sensory representations, decisions, attention) (Dosher & Lu, 2017). Especially when looking at the study by T. Zhang et al. (2010), which attributed a location transfer to a pre-test, one could assume that simply including a pre-test in our experiment and not the lack of object-specific learning enabled the transfer of the transsaccadic learning to the new locations. An argument against this is that in the studies of Valsecchi and Gegenfurtner (2016) and Valsecchi et al. (2020) a transfer to the opposite hemifield was found, as well, even though this location was not part of any pre-test.

One could also think of the transsaccadic learning as a process of forming object-specific Bayesian priors. Learning of stimulus-specific priors has been shown previously (Gekas, Chalk, Seitz, & Seriès, 2013; Kerrigan & Adams, 2013; Osterbrink et al., 2022), as well as the learning of multiple priors that are separated by spatial location (Nagai, Suzuki, Miyazaki, & Kitazawa, 2012) or symbolic visual cues (Petzschner, Maier, & Glasauer, 2012). Roach, Mcgraw, Whitaker, and Heron (2017) investigated how multiple highly specific priors are learned and when generalization across them occurs. They showed that subjects initially form a single prior by generalizing across distinct sensory signals. When sensory signals were coupled with different motor outputs, though, participants formed multiple priors. The authors suggested that the brain clusters sensory input into groups, and prior specificity may reflect that enough evidence is accumulated to justify clustering. A similar logic could be applied for transsaccadic learning. If there is sufficient evidence that there are different transsaccadic priors for certain objects, then separate specific priors are learned. Otherwise, a generalized prior is learned that is a lot faster and saves memory capacity.

### Generalization versus specificity

The learning of transsaccadic associations is tightly linked to object recognition. For the latter, translation invariance seems like an important property of the human visual system, although its automatic emergence has been brought into question (Kravitz, Vinson, & Baker, 2008). There are different ideas on how object recognition at varying positions is achieved, which are summarized in the review by Kravitz et al. (2008): Position-specific models suggest that multiple representations, each specific to an object at a particular location, are saved. By associating position-dependent representations of the same object in different positions, apparent position independence increases (Kravitz, Kriegeskorte, & Baker, 2010). In contrast, position-independent models propose the existence of object representations that are independent of retinal locations. In these models, visual input is transformed for comparison with these memorized representations.

Saving position-specific object representations or, in our case, location-specific transsaccadic associations is highly memory intensive. For recognizing objects in complex scenes it could be useful, though, because objects often appear in particular positions in in our visual field (Kravitz et al., 2008; Rousselet, Thorpe, & Fabre-Thorpe, 2004). Typical visual-field locations facilitate information propagation in the human occipital cortex (Kaiser & Cichy, 2018) and allow for a quicker object classification (within 140 ms) (Kaiser, Moeskops, & Cichy, 2018). In general, classification and object recognition can be obtained in about 100 to 200 ms (Rolls, Tovee, & Lee, 1991; Ullman & Soloviev, 1999), constraining the number of computational steps the brain can perform in this time (Carlson, Hogendoorn, Kanai, Mesik, & Turret, 2011). Especially during saccadic eye movements, fast processing for object recognition is therefore a mandatory prerequisite. This is fulfilled by position-specific learning that only uses simple feed-forward computations and a high degree of parallelism resulting in very fast processing (Ullman & Soloviev, 1999).

Learning position-independent transsaccadic associations (generalizing from old to new locations) is less memory intensive. Learning to recognize objects is thus possible with a much smaller training set (Han, Roig, Geiger, & Poggio, 2020). At the same time, because more complex transformations need to be employed, generalization is more computation intensive. And, the more complex the computations, the longer the processing time (Orchard, Martin, Vogelstein, & Etienne-Cummings, 2013), which might be disadvantageous for visual object recognition during saccades.

In our everyday life, we encounter vast numbers of objects at various positions. If there is a rule that is not specific to an object (e.g., a transsaccadic size transformation) that should be applied to all visual input, it is beneficial to generalize it from the learned location to other retinal locations, because it would save memory capacity. The only constraint would be that its physiological implementation has to be fast. Ward, Isik, and Chun (2018) showed that general transformations of object representations exist. If there are transsaccadic associations that are object specific, the learning should stay specific to that learning instance including the retinal location, because it allows a quicker and more precise recognition.

## Conclusions

Previous studies have shown that the visual system incorporates prior knowledge in the form of transsaccadic associations into peripheral perception. If objects are manipulated transsaccadically, their perception is biased in the direction of the transsaccadic change. But, there have been seemingly conflicting results on whether these learned transsaccadic associations are location specific or can be transferred to new locations. The current study thus investigated under which circumstances generalization occurs. Results showed that, when specific associations are learned for multiple objects, they are bound to the learned location. When only one transsaccadic change is learned for a single object, it generalizes to new locations. Learning at multiple locations, on the other hand, did not lead to generalization. Furthermore, generalization is also not specific to certain features such as size.

## Supplementary Material

Supplement 1
