# Supplementary material for: What determines location specificity or generalization of transsaccadic learning?
Source: J Vis. 2023 Jan 17;23(1):8. doi: 10.1167/jov.23.1.8 (PMC9851281; doi:10.1167/jov.23.1.8)
Supplement: Supplement 1 [file jovi-23-1-8_s001.pdf]

Supplemental Material

Table 1

Bayesian analysis of Experiment 1: model comparison

Model	BF_{10}	Error %
change direction	10.18973	±13.3%
object status	0.3720579	±0.95%
change direction + object status	3.42988	±3.31%
change direction + object status + change direction:object status	116.9584	±3.37%
position status	7.327911	±1.17%
change direction + position status	70.67187	±1.39%
object status + position status	2.783045	±1.36%
change direction + object status + position status	27.53537	±1.51%
change direction + object status + change direction:object status + position status	2343.354	±2.92%
change direction + position status + change direction:position status	24.13937	±2.16%
change direction + object status + position status + change direction:position status	9.288519	±3.67%
change direction + object status + change direction:object status + position status + change direction:position status	749.1936	±2.24%
object status + position status + object status:position status	3.604982	±2.66%
change direction + object status + position status + object status:position status	38.10718	±2.33%
change direction + object status + change direction:object status + position status + object status:position status	4418.262	±2.54%
change direction + object status + position status + change direction:position status + object status:position status	11.30387	±2.19%
change direction + object status + change direction:object status + position status + change direction:position status + object status:position status	1511.175	±5.57%
change direction + object status + change direction:object status + position status + change direction:position status + object status:position status + change direction:object status:position status	161320.6	±3.64%

Note: All models are compared to the null model that only includes the participant as a random factor. All models also include participant as a random factor.

Table 2

Bayesian analysis of Experiment 1: analysis of effects via the Inclusion Bayes Factor (model averaged)

Effects	P(prior)	P(posterior)	Inclusion BF
participant	1.00	1.00	NA
change direction	0.26	8.79e-04	9.94
object status	0.26	2.54e-04	0.383
object status:change direction	0.26	0.05	101.92
position status	0.26	0.01	18.58
position status:change direction	0.26	0.01	0.334
position status:object status	0.26	0.04	1.91
position status:object status:change direction	0.05	0.95	106.75

Table 3

Bayesian analysis of Experiment 2: model comparison

Model	BF_{10}	Error %
object status	1.168771	±1.97%
position status	1.053929	±0.73%
object status + position status	1.420171	±3.43%
object status + position status + object status:position status	0.4634681	±3.45%
change direction	0.8122364	±8.16%
object status + change direction	0.8720617	±1.85%
position status + change direction	0.7799825	±1.64%
object status + position status + change direction	1.021334	±1.86%
object status + position status + object status:position status + change direction	0.3650437	±4.99%
object status + change direction + object status:change direction	382.5833	±2.83%
object status + position status + change direction + object status:change direction	771.2292	±6.62%
object status + position status + object status:position status + change direction + object status:change direction	278.2527	±3.61%
position status + change direction + position status:change direction	0.244683	±2.24%
object status + position status + change direction + position status:change direction	0.3081368	±2.62%
object status + position status + object status:position status + change direction + position status:change direction	0.120725	±3.06%
object status + position status + change direction + object status:change direction + position status:change direction	220.5106	±3.9%
object status + position status + object status:position status + change direction + object status:change direction + position status:change direction	89.5676	±4.38%
object status + position status + object status:position status + change direction + object status:change direction + position status:change direction + object status:position status:change direction	193.2421	±6.06%

Note: All models are compared to the null model that only includes the participant as a random factor. All models also include participant as a random factor.

Table 4

Bayesian analysis of Experiment 2: analysis of effects via the Inclusion Bayes Factor (model averaged)

Effects	P(prior)	P(posterior)	Inclusion BF
participant	1.00	1.00	NA
object status	0.26	2.46e-03	1.23
position status	0.26	0.40	2.01
position status:object status	0.26	0.19	0.371
change direction	0.26	1.98e-03	0.754
change direction:object status	0.26	0.90	648.29
change direction:position status	0.26	0.16	0.295
change direction:position status:object status	0.05	0.10	2.16

Table 5

Bayesian analysis of Experiment 3: model comparison

Model	BF_{10}	Error %
test phase	0.2905364	±2.2%
position status	0.4933287	±1.18%
test phase + position status	0.1378786	±1.8%
test phase + position status + test phase:position status	0.06784707	±2.01%
change direction	0.7707902	±2.67%
test phase + change direction	0.2314443	±2.31%
position status + change direction	0.3777965	±3.02%
test phase + position status + change direction	0.1013098	±2.45%
test phase + position status + test phase:position status + change direction	0.04853962	±2.32%
test phase + change direction + test phase:change direction	30.92546	±2.4%
test phase + position status + change direction + test phase:change direction	19.34386	±3.86%
test phase + position status + test phase:position status + change direction + test phase:change direction	10.9332	±3.02%
position status + change direction + position status:change direction	0.1520682	±14.7%
test phase + position status + change direction + position status:change direction	0.03621487	±4.83%
test phase + position status + test phase:position status + change direction + position status:change direction	0.0189451	±6.72%
test phase + position status + change direction + test phase:change direction + position status:change direction	6.81231	±4.8%
test phase + position status + test phase:position status + change direction + test phase:change direction + position status:change direction	4.142604	±4.88%
test phase + position status + test phase:position status + change direction + test phase:change direction + position status:change direction + test phase:position status:change direction	1.994105	±15.37%

Note: All models are compared to the null model that only includes the participant as a random factor. All models also include participant as a random factor.

Table 6

Bayesian analysis of Experiment 3: analysis of effects via the Inclusion Bayes Factor (model averaged)

**Effects**	**P(prior)**	**P(posterior)**	**Inclusion BF**
SNo	1.00	1.00	
test phase	0.26	0.01	0.285
position status	0.26	0.26	0.616
position status:test phase	0.26	0.20	0.575
change direction	0.26	0.02	0.769
change direction:test phase	0.26	0.93	165.33
change direction:position status	0.26	0.14	0.362
change direction:position status:test phase	0.05	0.03	0.481
